# Extra-Abdominal Fibromatosis (Desmoid Tumor): A Rare Tumor of the Lower Extremity Arising from the Popliteal Fossa

**DOI:** 10.1155/2011/184906

**Published:** 2011-08-21

**Authors:** Mehmet Ali Kaygin, Ozgur Dag, Bilgehan Erkut, Azman Ates, Refik Cetin Kayaoglu, Hakan Kadioglu

**Affiliations:** ^1^Department of Cardiovascular Surgery, Erzurum Regional Training and Research Hospital, 25020 Erzurum, Turkey; ^2^Department of Cardiovascular Surgery, Medical Faculty of Atatürk University, Erzurum, Turkey; ^3^Department of Neurosurgery, Medical Faculty of Atatürk University, Erzurum, Turkey

## Abstract

Aggressive fibromatosis is a rare soft tissue tumor. Although it lacks metastatic potential, it can grow aggressively in a locally infiltrating pattern. The tumors frequently recur after surgical excision, which remains the treatment of choice. Optional combinations of radiotherapy and/or chemotherapy have been used postoperatively for recurrent disease and/or inoperable cases. A palpable mass was detected in the popliteal fossa of the right lower extremity in a 48-year-old man. Magnetic resonance imaging showed a contrast-enhancing noncalcified lesion initially felt to represent a vascular tumor. An invasive mass adherent to the surrounding tissue was visualized intraoperatively and extensively debulked. The patient's postoperative course was uneventful. Histologic examination of the surgical specimen was consistent with an extra-abdominal desmoid tumor. After appropriate recognition, wide local excision may be the most appropriate treatment for fibromatosis of the extremity. However, the rarity of this tumor and the difficulty inherent in distinguishing it from similar-appearing tumors are necessitating histologic confirmation of the diagnosis.

## 1. Introduction

Desmoid tumors, also known as aggressive fibromatosis, are an extremely rare entity. They are slow growing and histologically benign, but tend to be locally invasive at various anatomic sites. Desmoid tumors originate most frequently from abdominal fascial or musculoaponeurotic structures, although they may appear at extra-abdominal sites. The most common extra-abdominal locations include shoulder, chest wall, back, thigh, and head/neck [1, 2]. Extremity desmoid tumors are extremely rare.

Desmoid tumors pose a clinically challenging problem because of their tendency to mimic vascular neoplasms. Generally, histopathological examination is necessary for definitive diagnosis, as in our case. Radical resection is necessary for successful excision since desmoid tumors tend to recur locally. However, surgery, radiotherapy, or both are regarded as the treatment(s) of choice for lesions [3]. This case report documents an extremity desmoid tumor that had been diagnosed as a vascular tumor based on its magnetic resonance imaging (MRI) characteristics.

## 2. Case Presentation

**Figure 1:**
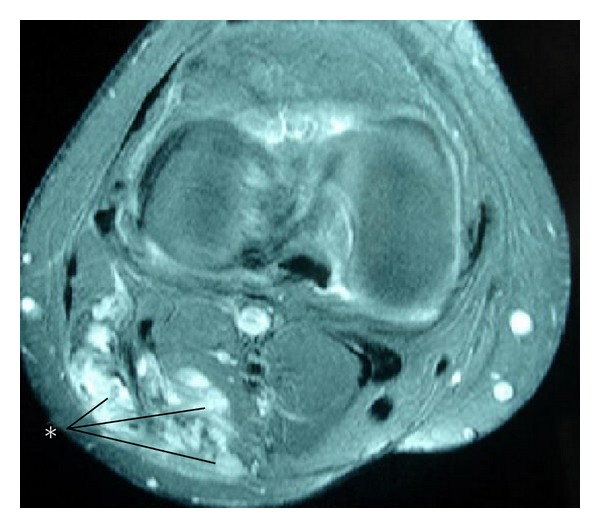
Axial image of MRI shows a large, expansive heterogeneous soft tissue mass with contrast, closely applied to the muscular structures, and infiltration and obliteration of adjacent structures (white asteriks).

A 48-year-old man presented with a tender swelling in his right lower extremity of 3 months' duration. Physical examination revealed a visually obvious 8 × 9 cm pulsatile mass on the lateral margin of the popliteal fossa. The distal extremity was stiff and tender to palpation. There was no previous history of trauma or surgical intervention. Lower extremity Doppler ultrasonography showed an 8 × 9 × 10-cm solid heterogeneous mass in the lateral popliteal fossa. Magnetic resonance imagining (MRI) demonstrated a large mass that closely approximated the muscular structures and surrounding connective tissue (Figure 1). The lesion was felt to represent a vascular mass.

**Figure 2:**
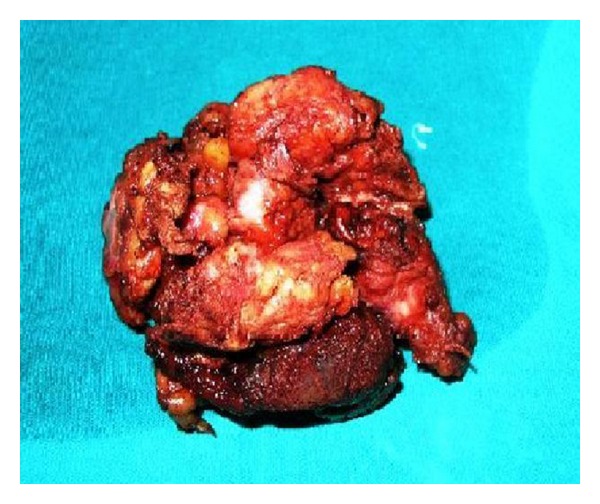
Gross cross-sectional view of pathologyic resected specimen. The gross lesion is poorly circumscribed and usually measures between 5 and 15 cm. On cut section, it is hard and tan-white. The lesion is poorly circumscribed and is centered in skeletal muscle and the adjacent fascia. There often are infiltration and obliteration of adjacent structures.

**Figure 3:**
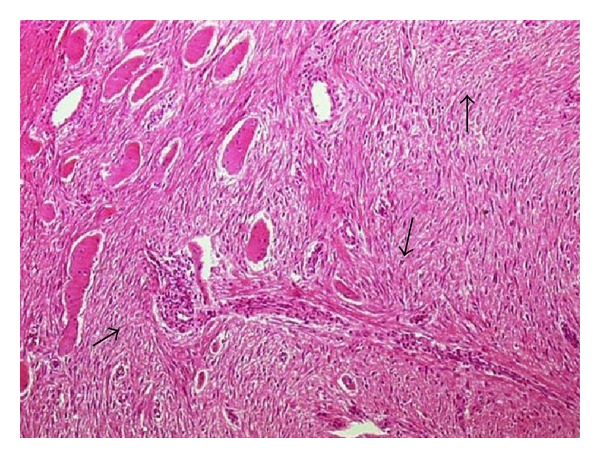
Desmoid fibromatosis showing fascicular arrangement of bland fibroblasts, which are interrupted by thin-walled, compressed vascular channels resulting in an appearance akin to a hypocellular scar. Note entrapped muscle fibers. No mitotic activity or nuclear pleomorphism is present (H&E, original magnification, 40x).

Intraoperatively, the patient was placed in the supine position under epidural anesthesia. The lesion was adherent to surrounding tissues including muscle and nerve. It originated from the muscular fascia of the deep muscle within the popliteal fossa. The lesion itself was large, gray-white, fibrotic, and irregular. Its appearance was not typical of a vascular lesion. No infiltration of the surrounding large vessels was identified, but the tumor invested nerves in the popliteal fossa. As this mass was thought to be suspicious for malignancy, enbloc dissection of the tumor was then carried out, including dissection of its attachments to the deep popliteal region, accomplished with the assistance of orthopedic and neurological surgeons. Total excision was attempted, but was unsuccessful as a tumor segment 0.5 × 0.5 cm in diameter which heavily infiltrated the tibial nerve could not be excised. Macroscopic surgical margins were free from all aspects of the tumor mass (Figure 2). Postoperatively, pathologic examination demonstrated widespread proliferation of spindle-shaped cells and collagen fibers. There were rare mitoses, but no signs of atypia were seen (arrow). Microscopic tumor margins were negative, and there was no evidence of malignant change (Figure 3).

The patient's early postoperative course was uneventful. The patient refused any subsequent radiotherapy or chemotherapy, and he was discharged on the 9th postoperative day. Eighteen months later, he is free from disease without evidence of local recurrence or distant metastasis.

## 3. Discussion

The incidence of desmoid tumors has been reported as 2–4 cases per 1 million [1, 2]. They are typically derived from the abdominal wall, the bowel and its mesentery, or in extra-abdominal sites such as chest wall, shoulder girdle, inguinal region, and neck [1–3]. Extra-abdominal desmoid tumors, as seen in our patient, very rarely originate from the extremities, and the only cases that have been previously documented in the popliteal fossa are mentioned in [3, 4]. 

Possible risk factors for the development of desmoid tumors include female sex, or a previous history of surgery, trauma, or pregnancy. Genetic susceptibility is believed to play a role in the development of the disease, but genetic risk factors have not been identified [5]. In our case, the patient had no identifiable risk factors for the development of a desmoid tumor. His mass was initially thought to be a vascular tumor but proved to be desmoid, highlighting the similar appearance of these two lesions on MRI.

Considering their rarity, desmoid tumors are often misdiagnosed. Prior studies, such as that of McKinnon et al. [6] noted that desmoid tumors were correctly diagnosed preoperatively in only 50% of cases. If clinical features are typical, the differential diagnosis typically includes vascular and soft tissue tumors such as fibrosarcomas or neurofibromas. The current case exhibited classical pulsatile swelling and pain, potentially consistent with the vascular etiology diagnosed by MRI, but actually representing a desmoid lesion. Despite of malignant tumors' view preoperatively, exact diagnosis was desmoid tumor in our case, histopathologically.

The histologic appearance of the tumor is usually consistent in various microscopic fields within a given case as well as from case to case. Desmoid tumors are described as dense, collagenous lesions with intertwining bundles of spindle cells without epithelial components. The tumor cells are uniform and lack mitotic activity. No necrosis or pleomorphism is present [7]. Grossly the tumor appears as a dense, hard, rubbery, grayish-white mass. It usually is a fixed tumor, and total resection often is impossible without compromising nearby structures, as encountered in our case.

In conclusion, desmoids arising in the lower extremity are extremely rare. They can mimic vascular tumors given their typical clinical and radiographic appearance. The possibility of an extremity desmoid tumor should be kept in mind when evaluating an extremity mass, but the diagnosis should be made only on the basis of a detailed histological examination. It is difficult to achieve complete resection because of their propensity for local invasion. For this reason, tumors should be removed as soon as possible after identification in order to achieve the most optimal resection possible.
